# Histopathological analysis of spontaneous large necrosis of adrenal pheochromocytoma manifested as acute attacks of alternating hypertension and hypotension: a case report

**DOI:** 10.1186/s13256-016-1068-3

**Published:** 2016-10-12

**Authors:** Nobumasa Ohara, Yasuyuki Uemura, Naomi Mezaki, Keita Kimura, Masanori Kaneko, Hirohiko Kuwano, Katsuya Ebe, Toshio Fujita, Takeshi Komeyama, Hiroyuki Usuda, Yuto Yamazaki, Takashi Maekawa, Hironobu Sasano, Kenzo Kaneko, Kyuzi Kamoi

**Affiliations:** 1 Department of Endocrinology and Metabolism, Nagaoka Red Cross Hospital, 2-297-1 Senshu, Nagaoka, Niigata 940-2085 Japan; 2 Department of Endocrinology and Metabolism, Uonuma Institute of Community Medicine, Niigata University Medical and Dental Hospital, Niigata, Japan; 3 Department of Cardiovascular Medicine, Nagaoka Red Cross Hospital, Niigata, Japan; 4 Department of Urology, Nagaoka Red Cross Hospital, Niigata, Japan; 5 Department of Pathology, Nagaoka Red Cross Hospital, Niigata, Japan; 6 Department of Pathology, Tohoku University Graduate School of Medicine, Miyagi, Japan; 7 Department of Internal Medicine, Ojiya General Hospital, Niigata, Japan; 8 Center of Diabetes, Endocrinology and Metabolism, Joetsu General Hospital, Niigata, Japan

**Keywords:** Pheochromocytoma, Hypertension, Hypotension, Immunohistochemistry, Adrenalectomy, Coagulative necrosis, Chest pain, Metaiodobenzylguanidine scintigraphy

## Abstract

**Background:**

Pheochromocytomas are rare catecholamine-producing neuroendocrine tumors. Hypertension secondary to pheochromocytoma is often paroxysmal, and patients occasionally present with sudden attacks of alternating hypertension and hypotension. Spontaneous, extensive necrosis within the tumor that is associated with catecholamine crisis is an infrequent complication of adrenal pheochromocytoma, but its pathogenesis remains unclear.

**Case presentation:**

A 69-year-old Japanese man developed acute-onset episodic headaches, palpitations, and chest pains. During the episodes, both marked fluctuations in blood pressure (ranging from 40/25 to 300/160 mmHg) and high plasma levels of catecholamines were found simultaneously. Radiological findings indicated a 4-cm left adrenal pheochromocytoma. These episodic symptoms disappeared within 2 weeks with normalization of plasma catecholamine levels. Two months later, the patient underwent adrenalectomy. Microscopic examinations revealed pheocromocytoma with a large central area of coagulative necrosis. The necrotic material was immunohistochemically positive for chromogranin A. Granulation tissue was adjacent to the necrotic area, accompanied by numerous hemosiderin-laden macrophages and histiocytes with vascular proliferation. Viable tumor cells, detected along the periphery of the tumor, demonstrated pyknosis, and the Ki-67 labeling index was 2 % in the hot spot. No embolus or thrombus formation was found in the resected specimen harboring the whole tumor. The Pheochromocytoma of the Adrenal gland Scaled Score was 2 out of 20. The patient’s postoperative course was unremarkable for > 7 years.

**Conclusions:**

Presumed causal factors for the extensive necrosis of adrenal pheochromocytoma in previously reported cases include hemorrhage into the tumor, hypotension induced by a phentolamine administration, embolic infarction, high intracapsular pressure due to malignant growth of the tumor, and catecholamine-induced vasoconstriction. In the present case, histopathological and clinical findings suggest that under conditions of chronic ischemia due to catecholamine-induced vasoconstriction, an acute infarction occurred after sudden attacks of alternating hypertension and hypotension. Over the subsequent 2 weeks, repetitive massive release of catecholamines from the infarcts into circulation likely accelerated infarction progression by causing repeated attacks of alternating hypertension and hypotension and resulted in the large necrosis. This case highlights the need for physicians to consider acute spontaneous tumor infarction accompanying episodic catecholamine crisis as a rare but severe complication of pheochromocytoma.

## Background

Pheochromocytomas are rare catecholamine-producing neuroendocrine tumors that arise from chromaffin cells of the adrenal medulla or extra-adrenal paraganglia [1]. The clinical presentation of pheochromocytoma, which is usually due to the direct actions of secreted catecholamines, is highly variable. Typical clinical presentation includes episodic headaches, palpitations, and hypertension. Hypertension is often paroxysmal, and patients occasionally present with sudden attacks of alternating hypertension and hypotension [2–4].

Spontaneous, extensive necrosis within the tumor that is associated with acute and severe clinical manifestations of massive catecholamine release, including acute abdomen, chest pain, or even shock, is an infrequent complication of adrenal pheochromocytoma [5–13]. However, the mechanisms underlying the development of such extensive necrosis remain unclear.

Here, we report a case of a patient who exhibited a large coagulative necrosis of an adrenal pheochromocytoma associated with acute attacks of alternating hypertension and hypotension, on which a detailed histopathological analysis was performed.

## Case presentation

A 69-year-old Japanese man was admitted to our hospital in July 2009 because of 1 week of episodic headaches, palpitations, and chest pains. He had a family history of type 2 diabetes mellitus (T2DM) in his mother. The patient drank 360 mL/day of sake. He had a smoking history of one pack of cigarettes/day from 20 to 60 years old. Our patient was diagnosed with T2DM at the age of 60 years at our hospital and started treatment with diet therapy (1600 kcal/day). He was started with the oral antidiabetic drug nateglinide (90 mg/day) at the age of 63 years, and subsequently had fair glycemic control with a mean glycated hemoglobin (HbA1c) of 7 % (reference range: 4.6–6.2 %) with no diabetic retinopathy. He had never had hypertension and had normal blood pressure (BP), with a systolic and diastolic BP of around 120 mmHg and 70 mmHg, respectively. He showed acute-onset episodic headaches, palpitations, and chest pains at rest in June 2009, which lasted for 10 to 20 minutes and occurred several times per day, and was admitted to our hospital.

On physical examination performed at presentation with no symptoms, he was 161 cm tall, weighed 55 kg, and his body temperature and blood pressure were 36.2 °C and 106/58 mmHg, respectively. No thyroid struma, chest rales, heart murmurs, abdominal tenderness or mass, or peripheral edema was detected. An electrocardiogram showed a normal sinus rhythm, with a heart rate (HR) of 65 beats/minute and no abnormal wave form. Chest X-ray showed no abnormalities with a cardiothoracic ratio of 46 %. Laboratory findings showed a normal complete blood count, normal liver and renal functions, normal balance of serum electrolytes, and high HbA1c value (7.6 %).

On day 2 of admission, our patient again experienced episodic headache, palpitation, and chest pain. During the episode, an electrocardiogram showed nonsustained ventricular tachycardia. His symptoms resolved within 20 minutes after sublingual administration of nitroglycerin (0.3 mg), which was administered as diagnostic therapy for ischemic heart disease, but it was unclear whether the drug was responsible for the observed improvement. Although an electrocardiogram did not show significant changes in the ST-T segment, the possibility of acute coronary syndrome remained because of his noticeable chest symptoms, and our patient received cardiac catheterization on the same day. Coronary arteriography showed no significant coronary artery lesion, and left ventriculography showed normal ventricular function with a left ventricular ejection fraction of 69 %. However, during the test, he again experienced episodic headache, palpitation, and chest pain accompanied by marked fluctuations of systemic BP (ranging from 40/25 mmHg to 300/160 mmHg). Catecholamine levels (noradrenaline 10.8 ng/mL, reference range: 0.10–0.50 ng/mL; adrenaline 8.8 ng/mL, reference range: 0–0.10 ng/mL) in his plasma taken during the attacks of alternating hypertension and hypotension were high. Neck, chest, and abdominal computed tomography (CT) performed following cardiac catheterization showed a 4-cm left adrenal tumor without any other mass lesion. He was believed to have possible pheochromocytoma, and treatment with an alpha 1 adrenergic blocker, oral doxazosin, (2 mg/day) was begun.

**Fig. 1:**
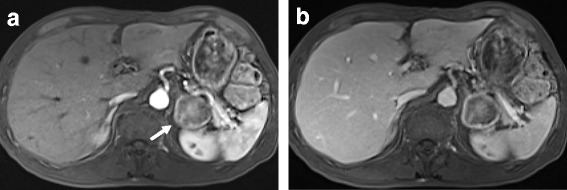
Magnetic resonance imaging (July 2009). Gadolinium-enhanced T2-weighted magnetic resonance imaging (**a**, early phase) indicated altered intensity in a 4-cm left adrenal tumor (*arrow*), and an area of high intensity was found along the periphery of the tumor, which persisted until the delayed phase (**b**)

Urinary excretion of catecholamines (noradrenaline 2592 μg/day, reference range: 26–230 μg/day; adrenaline 2032 μg/day, reference range: 1.0–29 μg/day) and metanephrines (normetanephrine 3.5 mg/day, reference range: 0.10–0.28 mg/day; metanephrine 6.0 mg/day, reference range: 0.04–0.18 mg/day) in a 24-hour urine sample collected from days 2 through 3 of admission were high. An iodine-123 metaiodobenzylguanidine (MIBG) scan detected no pathological accumulation in the patient’s whole body, including the region of the left adrenal gland. Gadolinium-enhanced T1-weighted magnetic resonance imaging (MRI) indicated altered intensity in a 4-cm left adrenal tumor, and an area of high intensity was found along the periphery of the tumor, which persisted until the delayed phase (Fig. 1a, b). Taken together, our patient was diagnosed with left adrenal pheochromocyroma containing a large necrosis. Our patient no longer experienced episodic headaches, palpitations, chest pains, or labile BP by day 5 of admission. Plasma levels of noradrenaline (0.38 ng/mL) and adrenaline (0.10 ng/mL) measured on the morning of day 7 were normal. In preparation for adrenal surgery [1], the dose of doxazosin was titrated to 12 mg/day, and he was discharged on day 19 after admission.

**Fig. 2:**
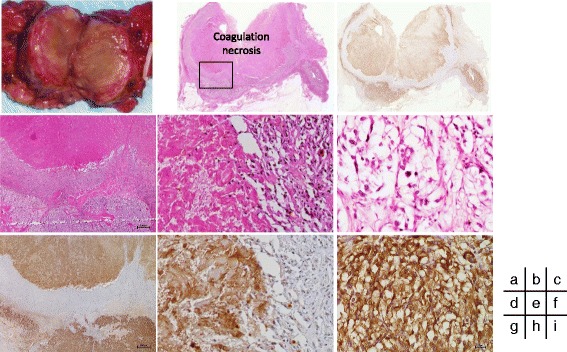
Histopathological findings of the resected left adrenal gland (September 2009). **a** Gross appearance of the cut surface of the left adrenal tumor 3 cm in size showed the inferior surface to be necrotic. **b−i** Microscopic examination of the left adrenal tumor (**b, d−f**; hematoxylin and eosin staining. **c, g−i**; chromogranin A staining). Nontumoral adrenal gland in the *right lower corner*, and well-encapsulated tumor in the remainder of the photograph (**b**). The tumor had a large area of coagulative necrosis in the center. The necrotic material contained morphologically ghost cells (**d, e**) and was immunohistochemically markedly positive for chromogranin A (**c, g, h**). There were numerous hemosiderin-laden macrophages and histiocytes accompanied by vascular proliferation in the region adjacent to the area of necrosis (**e, h**). The viable region along the periphery of the tumor contained numerous cells undergoing pyknosis (**f**), and the cytoplasm of the tumor cells was positive for chromogranin A staining (**i**)

Our patient underwent laparoscopic left adrenalectomy in September 2009. The histopathological features of the tumor were consistent with those of intra-adrenal paraganglioma (pheochromocytoma) [14] (Fig. 2). The tumor had a large area of coagulative necrosis in the center. The necrotic material was immunohistochemically positive for cytoplasmic components, such as synaptophysin and tyrosine hydroxylase, and was markedly positive for the neuroendocrine granule component, chromogranin A. Numerous hemosiderin-laden macrophages and histiocytes accompanied by vascular proliferation were detected in the region around the area of necrosis. The viable areas located at the periphery of the tumor contained numerous cells undergoing pyknosis, and the cytoplasm of these tumor cells was immunohistochemically positive for synaptophysin, tyrosine hydroxylase, and chromogranin A. The tumor cell nuclei were positive for SDHB. The Ki-67 labeling index was 2 % in the hot spot. The Pheochromocytoma of the Adrenal gland Scaled Score (PASS) [15] was 2 (two factors, nuclear pleomorphism and hyperchromasia, were met) out of a maximum score of 20. No embolus or thrombus formation, atherosclerosis, or vasculitis was found in the resected specimen harboring the whole tumor.

The patient discontinued oral doxazosin just after surgery and had no recurrent episodic headache, chest symptoms, or labile BP. His T2DM was treated with continued oral nateglinide, with a mean HbA1c of 7 %. His postoperative course during close follow-up for > 7 years has been uneventful without local or distant recurrence of pheochromocytoma.

## Discussion

An elderly Japanese patient exhibited spontaneous large necrosis of adrenal pheochromocytoma that manifested as 2 weeks of episodic headaches, palpitations, chest pains, and rapid fluctuations of hypertension and hypotension. Our patient underwent laparoscopic adrenalectomy 2 months after the episodes, and a diagnosis of pheochromocytoma was confirmed by immunohistochemistry. Light microscopic examination revealed a large coagulative necrosis in the tumor (Fig. 2). This finding was similar to those of previously reported cases of adrenal pheochromocytoma with a large central area of pure necrosis without hemorrhage or tumor rupture [5–7, 9–11]. In our patient, the necrotic material, containing morphologically ghost cells, yielded positive immunoreactivity for chromogranin A. Granulation tissue was adjacent to the necrotic area, accompanied by the presence of numerous hemosiderin-laden macrophages and histiocytes with vascular proliferation. Viable tumor cells were detected along the periphery of the tumor and demonstrated the presence of pyknosis. The viable tumor cells had a Ki-67 labeling index of 2 %, which did not necessarily indicate increased tumor proliferative activity. These findings suggest that the pheochromocytoma in our patient exhibited chronic ischemia due to abnormal vascular flow, and then acute infarction [16] occurred in the tumor resulting in the extensive necrosis.

Presumed causal factors for the extensive necrosis of adrenal pheochromocytoma in previously reported cases include hemorrhage into the tumor [8, 12, 13], hypotension induced by a phentolamine administration [5, 9], high intracapsular pressure due to malignant growth of the tumor [6], infarction secondary to vascular occlusion by an embolism [17], shock [10], and catecholamine-induced vasoconstriction of vessels supplying and/or within the tumor [7]. In the present case, no embolus or thrombus formation, atherosclerosis, or vasculitis was found in the resected specimen harboring the whole tumor. The sudden attacks of alternating hypertension and hypotension (with systolic BP ranging from 40 mmHg to 300 mmHg), accompanied by episodic headaches, palpitations, and chest pains, were simultaneously documented with a catecholamine burst during a cardiac catheter test. These findings suggest that under the conditions of chronic ischemia within the tumor due to catecholamine-induced vasoconstriction, a certain-sized acute infarction occurred, caused by sudden attacks of alternating hypertension and hypotension, in our patient. Subsequently, repetitive massive release of catecholamines from the infarcts into the circulation probably accelerated the progression of infarction by causing repeated attacks of alternating hypertension and hypotension accompanied by headaches, palpitations, and chest pains and resulted in the extensive necrosis within his pheochromocytoma.

Many previously reported cases of extensive necrosis in adrenal pheochromocytoma showed spontaneous remission of catecholamine crisis even before they underwent surgical removal of the tumor [5, 6, 12, 13]. In the present case, our patient’s acute-onset episodic headaches, palpitations, chest pains, and rapid fluctuations of hypertension and hypotension disappeared within 2 weeks with normalization of plasma catecholamine levels. These findings suggest that the large necrosis of our patient’s adrenal pheochromocytoma may have been completed over the 2 weeks.

Patients with extensive necrosis of adrenal pheochromocytoma often present with abdominal pain [5, 6, 12, 13, 17]. In the present case, the patient did not present with abdominal pain but exhibited episodic chest pains accompanied by headaches, palpitations, and rapid fluctuations of BP in the absence of evident cardiac disorder. Although patients with pheochromocytoma infrequently exhibit cardiac disorders causing chest pain, such as coronary artery disease and cardiomyopathy [18], cases of chest pain as an atypical manifestation of pheochromocytoma in the absence of a cardiac disorder have also been reported [19, 20]. Thus, although the precise mechanism remains unclear, our patient’s chest pain was probably due to intermittent massive release of catecholamines during the course of the development of the extensive necrosis of pheochromocytoma.

Most previously reported cases of spontaneous large necrosis of adrenal pheochromocytoma without rupture were shown to be benign [5, 7–13], but malignant cases have also occasionally been reported [6]. PASS is a histological scoring system used in clinical practice to separate benign from malignant pheochromocytomas by histomorphological parameters [15]. In this system, tumors with a PASS of 4 or more of 20 are considered to have potential for malignant behavior. Our patient exhibited a solitary unilateral adrenal pheochromocytoma that had a PASS of 2 and experienced no recurrence during postoperative follow-up for more than 7 years. Since recurrence of pheochromocytoma may occur even decades after surgery [21], further careful follow-up was needed in our patient.

Iodine-MIBG scintigraphy, in combination with anatomical imaging such as CT or MRI, has been used as a diagnostic tool for detecting chromaffin cell tumors in patients with biochemically confirmed pheochromocytoma [1]. The sensitivity of iodine123-MIBG scintigraphy for detecting pheochromocytoma is approximately 90 % [22], and known factors responsible for false-negative results include extra-adrenal or small adrenal pheochromocytoma, succinate dehydrogenase subunit B (SDHB) gene mutations, drug interference, and extensive necrosis in the tumor [1, 9, 23, 24]. In the present case, the extensive necrosis in his adrenal pheochromocytoma was probably responsible for the false-negative result on iodine123-MIBG scintigraphy.

## Conclusions

Histopathological and clinical findings suggest that under the conditions of chronic ischemia within the tumor due to catecholamine-induced vasoconstriction, an acute infarction occurred after sudden attacks of alternating hypertension and hypotension in our patient. Over the subsequent 2 weeks, a repeated massive release of catecholamines from the infarcts into the circulation probably accelerated the progression of infarction by causing episodic attacks of alternating hypertension and hypotension, resulting in the extensive necrosis of adrenal pheochromocytoma. This case highlights the need for physicians to consider acute spontaneous tumor infarction accompanying episodic catecholamine crisis as a rare but severe complication of pheochromocytoma.

## Abbreviations

BP: blood pressure
CT: computed tomography
HbA1c: glycated hemoglobin
HR: heart rate
MIBG: metaiodobenzylguanidine
MRI: magnetic resonance imaging
PASS: Pheochromocytoma of the Adrenal gland Scaled Score
SDHB: succinate dehydrogenase subunit B
T2DM: type 2 diabetes mellitus
